# SARS-Cov-2 Cycle Threshold Value and Olfactory Dysfunction in COVID-19 Patients

**DOI:** 10.22038/IJORL.2023.71462.3429

**Published:** 2023-09

**Authors:** Ali Goljanian Tabrizi, Maliheh Mohseni Ashjerdi, Matin Ghazizadeh, Majid Maleki

**Affiliations:** 1 *Department of Otolaryngology, Head and Neck Surgery, Shahid Beheshti University of Medical Sciences, Taleghani Hospital, Tehran, Iran.*

**Keywords:** COVID-19, Cycle threshold value, Olfactory impairment, SARS-CoV-2 infection, Coronavirus disease 2019, Olfaction disorders

## Abstract

**Introduction:**

Considering the inconsistent results regarding the association between the severity and duration of olfactory dysfunction (OD), and the viral load in coronavirus disease 2019 (COVID-19) patients, we aimed to conduct this study.

**Materials and Methods:**

This is a prospective cohort study in which COVID-19 patients were evaluated for the initial cycle threshold value (Ct values) measured by the nasopharyngeal samples along with olfactory function measured by the University of Pennsylvania Smell Identification Test (UPSIT) within 2 months of COVID-19 onset.

**Results:**

Among 309 COVID-19 patients who were included in this study, 108 (34.9%), 112 (36.2%) and 89 (28.8%) were normosmic, hyposmic, and anosmic, respectively based on the UPSIT. The severity of COVID-19 and the rate of hospitalization were higher in anosmic patients (p<0.0001, and p<0.0001, respectively). Moreover, significant associations between the initial Ct value and the severity of OD at admission and follow-ups were detected (p<0.0001 and p<0.0001, respectively). Anosmic patients had higher Ct values in comparison with hyposmic (approx. 3-fold) and normosmic (approx. 12-fold) patients. The recovery rate after one- and two-month follow-ups was 47% and 84%, respectively. At the follow-ups, OD-recovered patients significantly had lower Ct values (mean Ct value: 27.79 ± 2 and 28.21 ± 2.08) in comparison with those who have not recovered yet (mean Ct value: 30.19 ± 3.36, and 33.6 ± 3.37) (p<0.0001, and p<0.0001, respectively).

**Conclusions:**

Ct value seems to be a significant factor not only in predicting OD severity in COVID-19 patients but also in the OD recovery duration. This finding may be helpful to investigate the underlying mechanisms of OD in COVID-19 patients.

## Introduction

The 2019 outbreak of novel coronavirus disease (COVID-19) has posed a significant threat to global public health which was caused by the severe acute respiratory syndrome 2 virus (SARS-CoV-2 virus) (1,2). In addition to the commonly recognized symptoms of COVID-19 such as fever, sore throat, dyspnea, cough, and expectoration which were widely used to monitor suspicious patients with positive contact or travel histories (3), acute olfactory dysfunction (OD) has emerged as a prominent feature of COVID-19. The evidence discussed the promising use of olfactory function assessment in the diagnosis of COVID-19 (4-7). Considering the high prevalence of chemosensory dysfunction (68–85.6%) among COVID-19 patients (8-10), the American Academy of Otolaryngology-Head and Neck Surgery has persuaded to include anosmia and hyposmia in the list of diagnostic markers for susceptible COVID-19 infection (11). The SARS-CoV-2 virus has been found to disrupt the olfactory pathway, with over 70% of patients reporting OD (12-17). 

The diagnosis of COVID-19 is established when the viral nucleic acid is detected by real-time reverse transcriptase polymerase chain reaction (RT-PCR), in either the upper respiratory specimens via nasopharyngeal or oropharyngeal swabs or lower respiratory samples (18). RT- PCR generates a cycle threshold (Ct) value, which is explained by the number of amplification cycles required to meet a threshold for the identification of the viral nucleic acid. The target virus concentration in the specimen is inversely associated with the Ct level. Therefore, the lower the Ct value are, the higher the viral load and the level of viral replication activity are presented (19). 

The relationship between Ct values and the severity of COVID-19 remains unclear. Based on a literature review, it has been reported that more severe patterns of COVID-19 infection are associated with a higher viral load of SARS-CoV-2 (20,21). 

In contrast, Zou et al. found no significant difference in the viral load between asymptomatic and symptomatic individuals (22). These discrepancies could be attributed to the variation in the study design, the time of sampling, and the method of collecting the respiratory specimen. Whether this potential relationship is also valid for OD requires further investigation. There have been inadequate surveys on patients with COVID-19 to understand the correlation between OD severity and viral load kinetics. To the best of our knowledge, only a few studies have already evaluated the correlations between viral load and severity of and recovery from chemosensory dysfunction directly in small populations (23,24). Considering the conflicting results, we aimed to investigate the association between viral load and the severity of OD in patients affected by COVID-19. Moreover, we tested the hypothesis that whether the recovery from chemosensory dysfunction in COVID-19 patients is correlated to their viral load.

## Methods and Materials

This observational cohort study was carried out at Ayatollah Taleghani Hospital between February and June 2021. We enrolled hospitalized adults with confirmed COVID-19 infection through PCR testing. Patients with a pre-existing history of OD before the pandemic, previous nasal surgery or radiotherapy, chronic rhinosinusitis, allergic rhinitis, head and neck trauma, a history of psychiatric or neurological disorders, and non-cooperative patients were excluded from the study.

*Ethical considerations*

Written informed consent was obtained from all the patients. No patients were obligated to participate in the study and patients were not precluded from treatments in case they did not participate in the study. 

The study was conducted in accordance with the ethical consideration of the ethics committee of the Shahid Beheshti University of Medical Sciences (IR. SBMU. MSP. REC. 1401.071) and the Helsinki Declaration of 1964 and its later amendments.

*Study design*

Based on clinical symptoms, an emergency physician evaluated suspected patients in terms of COVID-19 infection, and the RT-PCR test confirmed the COVID-19 disease. Nasopharyngeal samples were obtained by trained personnel from patients who presented symptoms suggestive of COVID-19 upon hospital admission.  The samples were transferred to the hospital's virology laboratory. RT-PCR was performed via Sansure Biotech's 2019nCoV 30Minute Nucleic Acid Reagent Kits (Sansure Biotech, Inc., Development Zone, Changsha, China), and the Roche light cycler. CT values were recorded for each patient.

The participants information was collected using a pre-defined checklist. The information collected at admission included demographic characteristics such as age, gender, and BMI, clinical information including previous medical histories such as hypertension and diabetes, the onset of COVID-19 symptoms, the onset of OD, the severity of OD (subjective and objective), the viral load based on the cycle threshold of the RT-PCR test, and the severity of COVID-19 symptoms using the COVID-19 symptom index (CSI) (25). The CSI assigns a score ranging from 0 (no problem) to 4 (very serious problem) for the severity of 25 common COVID-19 symptoms. Therefore, the total score can range from 0 (no symptoms) to 100 (very severe symptoms). In addition, patients were followed up two months after the initial examination, and the severity of OD was assessed using the objective University of Pennsylvania Smell Identification Test (UPSIT).

*Olfactory evaluation*

To accurately measure olfactory function, a modified Persian version of the UPSIT (Sensonics International, Haddon Heights, NJ) was used (17,26).  This self-administrated 40-odorant test has been validated and proven to be reliable (test-retest, r = 0.94) (27).  This multiple-choice test helps classifying test results into meaningful functional categories, such as anosmia, hyposmia, and normosmia. The hospital-based olfactory examination was performed by the senior author. Following the completion of the hospital testing, each patient was given a UPSIT with a detailed instruction manual of the test to self-administer at home. The patients were then contacted by phone calls to confirm their willingness to undergo follow-up testing at the appropriate time for retesting. Patients were instructed to avoid food and beverages for 15 minutes before the smell test. Each patient returned a photograph of their selections for each of the 40 odorants to calculate their score.

*Statistical analysis*

 SPSS 26.0 was used to perform the statistical analysis (IBM, Armonk, NY). Categorical variables are reported as counts and percentages. The mean and standard deviation are provided as descriptive statistics for quantitative variables. The ANOVA test with Tukey post-hoc was conducted to evaluate the statistical significance of differences in continuous variables, such as cycle thresholds, across OD severity levels. Student T-test was used to compare Ct values between OD severity categories when OD was categorized as two separate groups of OD and normosmia. The Chi-square test was used to assess the significance of differences in categorical variable distribution across OD severity levels. Pearson's correlation coefficient was used to assess the relationship between subjective and objective OD responses. The statistical significance threshold was considered at P 0.05.

## Results

In this study, we enrolled 309 patients with confirmed COVID-19 including 229 (74.11%) females and 80 (25.89%) males. The mean age of the participants was 38.81 ± 11.16 years and their mean BMI was 26.49 ± 5.78 kg/m^2^. Further clinical characteristics of the participants were shown in Table 1. In addition, these characteristics were stratified by the status of OD of patients in three groups (Anosmia, hyposmia, and normosmia). Patients with anosmia were younger than patients with normosmia (p= 0.008). There were no significant differences in terms of gender,BMI,smoking, and hypertension across different OD groups (Table 1).

**Table 1:** Background demographic and clinical characteristics of the participants

	**Anosmia (n=89)**	**Hyposmia (n=112)**	**Normosmia (n=108)**	**Total (n=309)**	**P value ** ^a^
Age (years) [mean (SD)]	35.92 (10)	39.15 (10.92)	40.82 (11.89)	38.81 (11.16)	0.008
Gender [male (%)]	30 (33.71)	22 (19.64)	28 (25.93)	80 (25.89)	0.078
BMI (kg/m^2^) [mean (SD)]	26.25 (6.15)	26.88 (6)	26.29 (5.25)	26.49 (5.78)	0.675
Smoking [Yes (%)]	10 (11.24)	12 (10.71)	15 (13.89)	37 (11.97)	0.744
Hypertension [Yes (%)]	13 (14.61)	13 (11.61)	20 (18.52)	46 (14.89)	0.353
Diabetes [Yes (%)]	0 (0)	10 (8.93)	2 (1.85)	12 (3.88)	0.002

^a ^One-way ANOVA

Information on the clinical aspects of the course of COVID-19 in the participants is presented in Table 2. On average, in patients with anosmia and hyposmia, OD often manifested 1.47 and 2.42 days after the onset of other COVID-19 symptoms, respectively. Patients without OD were referred to the hospital later than those with either form of OD (anosmia or hyposmia) (p<0.0001). COVID-19 was generally more severe in patients with anosmia compared to patients with hyposmia or normosmia, In other words, all the critically ill patients were anosmic (p<0.0001). Patients with anosmia had a higher score of COVID-19 severity index and longer hospitalization compared with patients with hyposmia and normosmia (p<0.0001 and p<0.0001, respectively). Blood parameters including CRP, WBC, and lymphocyte percentage did not differ substantially across the three groups. All in all, all patients with normosmia recovered from COVID-19, however, the rate of recovery was significantly lower in patients with hyposmia and anosmia (p<0.0001) (Table 2). 

**Table 2:** Clinical characteristics regarding the course of COVID-19 infection among participants

		**Anosmia (A)**	**Hyposmia (B)**	**Normosmia (C)**	**Total**	**P value ** ^a^	**Significant comparisons**
The onset of olfactory dysfunction ^b^ [mean (SD)]		1.47 (3.02)	2.42 (2.94)	N/A	1.91 (3.01)	0.03	
Hospitalization day ^c^ [mean (SD)]		9.03 (6.47)	8.78 (6.93)	16.38 (12.33)	11.31 (9.57)	<0.0001	A-C, B-C
COVID-19 severity	Mild	1 (1.12)	8 (7.14)	35 (32.41)	44 (14.24)	<0.0001	
	Moderate	56 (62.92)	81 (72.32)	67 (62.04)	204 (66.02)		
	Severe	26 (29.21)	23 (20.54)	6 (5.56)	55 (17.8)		
	Critical	6 (6.74)	0 (0)	0 (0)	6 (1.94)		
COVID-19 severity index [mean (SD)]		49.64 (17.86)	42.03 (11.8)	31.5 (12.18)	40.54 (15.69)	<0.0001	A-B, B-C, A-C
CRP [mean (SD)]		22.96 (29.03)	18.13 (19.21)	17.31 (19.81)	19.3 (22.84)	0.2	
Lymph (%) [mean (SD)]		27.75 (11.28)	29.67 (11.17)	28.64 (10.65)	28.75 (11.02)	0.47	
WBC [mean (SD)]		4.91 (1.81)	4.86 (2.17)	5.22 (1.89)	5 (1.98)	0.349	
Days of hospitalization [mean (SD)]		18.9 (11.92)	13.26 (6.82)	10.23 (5.22)	13.97 (8.97)	<0.0001	A-B, B-C, A-C
Progression	Recovery	67 (72.58)	100 (89.29)	108 (100)	275 (89)	<0.0001	
	ICU admission	10 (11.24)	12 (10.71)	0	22 (7.12)		
	Intubation	3 (3.37)	0	0	3 (0.97)		
	Death	9 (10.11)	0	0	9 (2.91)		

^a ^One-way ANOVA with Tukey post-hoc, ^b^ Days after the onset of other COVID-19 symptoms, ^c^ Number of days after onset of COVID-19 symptoms

Based on the UPSIT test, from the 309 patients diagnosed with COVID-19 at hospital admission, 108 of them were normosmic, 112 of them had some degrees of hyposmia and 89 of them were anosmic. Meanwhile, according to self-reported records, 78, 35, and 101 were normosmic, hyposmic, and anosmic, respectively. There was a significant direct correlation between objective and subjective (self-reported) tests of OD at the hospital admission (B= 0.833, p<0.0001) (Table 3). 

**Table 3:** Subjective and objective olfactory dysfunction status of patients at admission, one and two months after

	**At admission**			**At 30 days**			**At 60 days**		
	Normosmia	Hyposmia	Anosmia	Normosmia	Hyposmia	Anosmia	Normosmia	Hyposmia	Anosmia
Normosmia	73	5	0	78	0	0	78	0	0
Mild hyposmia	15	43	0	49	9	0	58	0	0
Moderate hyposmia	11	24	0	31	4	0	35	0	0
Severe hyposmia	9	28	0	32	5	0	37	0	0
Anosmia	0	12	89	12	57	32	69	26	6
Total	108 (34.95)	112 (36.25)	89 (28.8)	202 (65.37)	75 (24.27)	32 (10.36)	277 (89.64)	26 (8.41)	6 (1.94)

**Fig 1:**
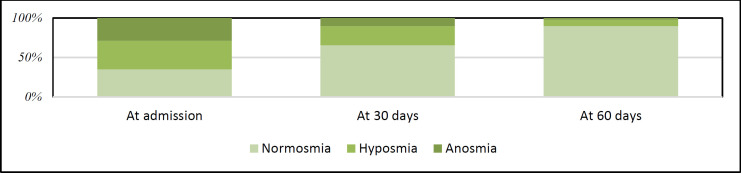
Objective olfactory dysfunction status of patients at admission, one and two months after

Objective evaluation of OD after 30 and 60 days showed gradual recovery of OD. At admission, 201 (65.05%) of patients had OD, which declined to 108 (34.63%) and 32 (10.35%) of patients after 30 and 60 days, respectively. As demonstrated in Table 2, all the patients with reported normosmia, were normosmic after one month, however, patients who reported variable degrees of hyposmia got normosmic after 2 months. About 12 and 70 percent of patients with self-reported anosmia at admission recovered from OD at 1 month and 2 months, respectively (Figure 1).

Table 3 shows the Ct values across groups with different OD status at admission, one and two months after admission. The mean Ct values among groups that were stratified based on subjective self-reported OD at admission showed a significant difference. Patients with self-reported anosmia had higher Ct values, therefore lower viral load, in comparison with other groups (p<0.0001). 

Likewise, a comparison of Ct values according to objective OD severity at admission gave similar results, in which anosmic patients had higher Ct values and lower viral load in comparison with hyposmic (approx. 3-fold) and normosmic (approx. 12-fold) patients. Moreover, hyposmic patients also had higher Ct values and lower viral load (approx. 4-fold) in comparison with normosmic patients (Table 4).

**Table 4:** Ct values from the SARS-CoV-2 polymerase chain reaction assay by the olfactory dysfunction tests at admission, one and two months after

**Timepoint**			**Ct**	**P value ** ^a^	**Significant comparisons**
At admission	Subjective OD severity at admission [mean (SD)]	Anosmia (A)	29.31 (3.77)	<0.0001	A-B, A-C, A-D, A-E
		Severe hyposmia (B)	27.61 (2.54)		
		Moderate hyposmia (C)	26.99 (2.62)		
		Mild hyposmia (D)	27.67 (2.63)		
		Normal (E)	27.58 (2.64)		
	Objective OD severity at admission [mean (SD)]	Anosmia (A) (n=89)	29.92 (3.59)	<0.0001	A-B, A-C, B-C
		Hyposmia (B) (n= 112)	28.4 (2.36)		
		Normosmia (C) (n=108)	26.29 (2.44)		
One month	Objective OD severity at 30 days [mean (SD)]	Anosmia (A) (n=32)	33.61 (3.38)	<0.0001	A-B, A-C, B-C
		Hyposmia (B) (n=75)	28.74 (2.06)		
		Normosmia (C) (n=202)	26.99 (2.37)		
Two months	Objective OD severity at 60 days [mean (SD)]	Anosmia (A) (n=6)	39.94 (0.07)	<0.0001	A-B, A-C, B-C
		Hyposmia (B) (n=26)	32.15 (1.52)		
		Normosmia (C) (n=277)	27.46 (2.42)		

^a ^One-way ANOVA with Tukey post-hoc

As it is demonstrated in Table 3, this pattern persisted over time after one and two months. The Ct values of patients with anosmia at admission (n=89) were lower than the patients with anosmia after one and two months.

To assess the association of Ct values with OD recovery, we analyzed the patients with OD (including anosmia and hyposmia) respecting recovery (being normosmic) after one and two months. The recovery rate after one and two months was approximately 47% and 84%, respectively. Patients who recovered from OD after one month had significantly lower Ct values and higher viral load in comparison with patients who still had OD (p<0.0001). Similarly, patients who recovered from OD after two months had significantly lower Ct values and higher viral load in comparison with patients who still had OD after two months (p<0.0001) (Table 5).

**Table 5:** Comparison of Ct values in patients with or without recovery on the 30^th^ or 60^th^ day who had OD at admission

**At admission**	**At 30** ^th^ ** day**	**Ct**	**P value ** ^a^	**At 60** ^th^ ** day**	**Ct**	**P value ** ^a^
OD (n=201, 65.05%)	Normal (n=94)	27.79 (2)	<0.0001	Normal (n=169)	28.21 (2.08)	<0.0001
	OD (n=107)	30.19 (3.36)		OD (n=32)	33.6 (3.37)	

^a ^Student T-test

## Discussion

In this cohort study, we aimed to investigate the association between OD and recovery from it with SARS-CoV-2 viral load and COVID-19 disease severity. OD typically appeared 2 days after the onset of other COVID symptoms with approximately 65 percent of confirmed COVID-19 patients experiencing OD ranging from mild hyposmia to anosmia. The objective evaluation of OD was closely correlated with self-reported OD. Patients who did not develop experience OD were referred and admitted significantly later than those who did, which may be attributed to the concerning effect of losing the smelling sense. Moreover, patients with anosmia experienced more severe disease, longer hospitalization, a lower rate of recovery from COVID-19, and higher risk of mortality. Patients with OD, either anosmia or hyposmia, had significantly higher Ct values and subsequently lower viral load when compared with normosmic patients. 

Due to the substantial evidence that suggests OD may be a significant manifestation of COVID-19 infection, researchers have focused more on the underlying pathophysiology and prognosis of this chemosensory dysfunction. The etiology of OD has not been completely known yet (28,29). During the early stages of the COVID-19 pandemic, some researchers linked OD to the neuro-invasion and death of the olfactory bulb neurons (30,31). However, recent well-designed studies have documented that the olfactory epithelium is the target of viral invasion contributing to OD. Short recovery time, development of OD in mild COVID-19 cases, upregulation of the ACE-2 receptors as viral receptors on the olfactory epithelium, and presence of tissue damage and olfactory cleft edema in anosmic patients supports the theory of viral invasion to olfactory epithelium rather than irreversible neuronal invasion (32-41). 

Primary investigations with long-term monitoring revealed a considerable frequency of OD among infected patients and also persistent severe OD, ranging from 5 to 11 percent of cases (42-45). This implies that -the long-lasting OD related to COVID-19could pose a significant public health issue. Neither risk factors contributing to developing OD nor protecting factors attributed to recovering from OD in COVID-19 patients are well understood (12,14,17,46,47). In addition, identifying the risk factors related to persistent OD is crucial. The most current theory regarding the cause of this condition suggests that the infectious viral load may be a crucial factor. 

To test this hypothesis, we aimed to conduct a cohort study to investigate whether viral load plays a role in the severity of OD in COVID-19 patients. We also monitored patients for two months to determine whether there is a correlation between the initial viral load and recovery from OD in anosmic patients. Overall, our findings revealed a significant relationship between Ct values and OD severity in COVID-19 patients, meaning that higher Ct values were associated with greater likelihood of OD. This means that viral load is inversely associated with the severity of OD. Moreover, we demonstrated that patients who recovered from OD after one or two months had significantly lower Ct values in comparison with patients with persistent OD after this time frame. 

To the best of our knowledge, there are not many research on this topic and just few studies currently have been published in this regard. These studies have the merit of being the first to explore the potential associations between viral load and the severity of OD or OD recovery duration in COVID-19 patients. In a retrospective cohort study on 599 outpatient cases, the association between the severity (anosmia and hyposmia), and recovery time of OD with Ct values of SARS-CoV-2 were evaluated. They found that the mean Ct values in patients with anosmia were considerably greater than those with hyposmia (p=0.02). However, there was no significant difference in mean Ct values between patients who fully recovered from OD and those who did not (p=0.62). Among the 83 COVID-19 patients who were recruited in a prospective cohort study in China, although the mean level of Ct values in anosmic patients was higher than those who have normosmia, the difference was not significant (23). On the other hand, in a study including 288 COVID-19 patients with OD, no significant correlation between viral load and COVID-19 severity or poor olfactory outcome was detected. The study also revealed that clinical markers did not predict recovery from olfactory dysfunction during the two-month follow-up period. However, they indicated that lower levels of salivary and nasal antibodies, but not the serum antibodies, are related to poor olfactory outcomes within two months. This suggests a key role of local immune responses in OD-associated COVID-19 (48). Jain et al reported that COVID-19 patients with olfactory and taste dysfunction had a lower Ct value, and hence, a higher viral load at diagnosis (49).

In concordance with the result of this study, Vaira et al. showed that the correlation between viral load and olfactory scores at baseline (p = 0.844) and two-month follow-up (p = 0.519) was weak and not significant among the 60 included COVID-19 patients. The study suggests that individual factors may contribute to olfactory dysfunction rather than viral load or activity (24). Additionally, there was no significant difference between partially recovered patients and completely recovered patients (p=0.38) (50). several reasons could explain these conflicting results. First, different studies used different methods, which can lead to inconsistent results. Second, failure to use an objective assessment of olfactory function can result in recall bias. Furthermore, in some studies, self-reported OD has been shown to underestimate the actual prevalence of OD (51,52), hampering to draw a clear conclusion from the collected data. Third, population disparities that have been observed in other COVID-19 studies might be underscored. In addition, variations in study results can also be attributed to differences in sample size and type of sampling. Finally, COVID-19 detection through the use of PCR tests can vary depending on the kit, instruments, and operator. Also, the COVID-19 PCR test's variable false negative rate may confound the recruitment process (53). It is worth noting that several studies have suggested that Ct values should not be considered a reliable indicator for estimating viral load; thus, the indirectly calculated viral load based on Ct should be considered with great caution (54,55). 

## Conclusion

In this prospective cohort study among 309 COVID-19 patients, we found a significant association between the Ct value and the severity of OD which was measured by a valid and reliable self-reported test. Moreover, in patients who developed OD, a significant correlation between the 1-month and 2-month OD recovery and the initial Ct value was detected. Ct value seems to be a crucial factor not only in predicting OD development in COVID-19 patients but also in the OD recovery duration. 

Further studies are needed to confirm our findings and explore other potential factors contributing to OD in COVID-19 patients. Given the potential long-term consequences of chronic OD, identifying risk factors related to persistent OD is crucial. Treatments with the mechanism of reducing viral load should be assessed for their efficacy in modulating COVID-19-associated OD in the future clinical trials. 
